# The protective roles of tea tree oil extracts in bovine mammary epithelial cells and polymorphonuclear leukocytes

**DOI:** 10.1186/s40104-020-00468-9

**Published:** 2020-06-15

**Authors:** Kang Zhan, Tianyu Yang, Baobao Feng, Xinyu Zhu, Yinyin Chen, Yongjiu Huo, Guoqi Zhao

**Affiliations:** 1 Institute of Animal Culture Collection and Application, College of Animal Science and Technology, Yangzhou University, Yangzhou, 225009 China; 2 Wuxi Chenfang Biotechnology Co., Ltd., Wuxi, China

**Keywords:** Bovine mammary epithelial cells, Mastitis, PMNL, *Staphylococcus aureus*, Tea tree oil

## Abstract

**Background:**

Tea tree oil (TTO) plays an important role in antibacterial activity and alleviating the inflammatory responses. Bovine mammary epithelium and polymorphonuclear leukocytes (PMNL) can actively respond to bovine mastitis infection. However, regulatory effects of TTO extracts on the innate immune response of bovine mammary epithelial cells (BMECs) and PMNL remain not reported. Therefore, aim of the study was to evaluate the effects of TTO extracts on the mRNA levels of the genes involved in the innate immune response of BMECs and PMNL.

**Results:**

Our results demonstrated that addition of 0.025% and 0.05% TTO increased the proliferation of BMECs, and significantly enhanced (*P* < 0.05) the viability of BMECs exposed to *Staphylococcus aureus* (*S. aureus*). An inhibitory effect was observed against the growth of *S. aureus* by TTO incubation. The 0.05% TTO reduced *S. aureus* biofilm formation, association and invasion of *S. aureus* to BMECs, and changed the morphological and structural features of *S. aureus*. The proinflammatory cytokines IL-1β, IL-6, and TNF-α were decreased (*P* < 0.001) by the incubation of TTO. Interestingly, the expression of IL-8 known for PMNL chemotactic function was elevated (*P* < 0.05) by 0.05% TTO treatment. Consistently, 0.05% TTO increased the migration of PMNL in *S. aureus*-exposed BMECs when compared with *S. aureus* treatment alone (*P* < 0.05). In addition, PMNL incubated with 0.05% TTO decreased the levels of NFKB inhibitor alpha (NFKBIA) and TNF-α.

**Conclusions:**

Our results indicate that use of TTO can relieve the BMECs pro-inflammatory response caused by *S. aureus* and promote the migration of PMNL to mount the innate immune responses, and it may be novel strategy for the treatment of bovine mastitis caused by *S. aureus*.

## Introduction

Bovine mastitis, an inflammation of mammary gland, is a serious infectious disease involved in the infection with a pathogen, such as *Staphylococcus aureus* (*S. aureus*), *Escherichia coli*, and *Streptococcus* [1], and results in decreased milk quality [2] and economic losses [3]. Bovine mastitis is divided into clinical and subclinical mastitis. Clinical mastitis is readily observed and easily detected by abnormal milk secretions, whereas subclinical mastitis is asymptomatic and difficult to be observed and is diagnosed by the reservoir of pathogen infection in milk [4]. Recent investigation showed that *S. aureus* is a major cause of contagious bovine subclinical mastitis [5]. However, the emergence of antibiotic resistant strains of *S. aureus* known for methicillin-resistant *S. aureus* (MRSA) is a serious health hazard around the world [6], and MRSA infections have been observed in cases of bovine mastitis [7].

Some antibiotics to cure the clinical bovine mastitis have been widely used in the past study. However, many consumer concerns about the development of resistance to antibiotics agents are worried in some developing countries [8]. Furthermore, antibiotics to treat the bovine mastitis triggered by *S. aureus* has poor efficacy due to a low cure rate in clinical mastitis [9]. Importantly, the treatment of bovine mastitis using antibiotics is worried for human health because of the presence of residues of these antibiotics in milk. Therefore, the development of novel, alternative therapies strategies to both inhibit the pathogen and have the capacity to mount the innate immune response to kill the pathogen are urgent need for improving milk quality and human health.

Many plant extracts and essential oils derived from some plants contain secondary metabolites that shows antibacterial activity properties [10, 11]. Tea tree oil (TTO), an essential oil extracted from the leaves of *Melaleuca alternifolia* (*M. alternifolia*), plays an important role in antibacterial activity and anti-inflammatory properties [12]. TTO extract from the leaves of *M. alternifolia* contains the terpinen-4-ol, γ-terpinene, α-terpinene, 1,8-cineole, and α-terpineol [13]. The antibacterial activity of TTO primarily depends on its ability to result in increased membrane permeability, and it also disrupts the bacterial cell membrane structures and function [14, 15]. In addition, previous studies have investigated that innate immune responses play an important role in the resistance to pathogen [16] and improvement of somatic cells in milk [17]. During bovine mastitis, recruitment of circulating blood PMNL to mammary gland for mounting the innate immune responses are critical for alleviating the inflammatory response of bovine mammary epithelial cells (BMECs) and resolution of mastitis [18]. However, to our knowledge, no study has investigated the effects of TTO on the innate immune response of BMECs and PMNL function. We hypothesized that TTO can alleviate the proinflammatory responses of BMECs exposed with *S. aureus* and mount the innate immune responses of BMECs and PMNL.

Therefore, the objectives of this study were to evaluate the effects of TTO extracts on the expression of genes involved in the inflammatory response of BMECs exposed with *S. aureus*, the innate immune response of BMECs and PMNL, and the chemotactic ability of PMNL.

## Materials and methods

### Preparation of tea tree oil extracts

Tea tree essential oil preliminary product were obtained from True Blue Organics (New Zealand). The TTO extract was carried out by Wuxi Chenfang Biotechnology Co., Ltd. (Wuxi, Jiangsu, China). The oil preliminary product was loaded into reaction still. Then, the nitrogen was added into the reaction still. In addition, reaction still continuously maintains the 0.2 kg/cm^2^–1 kg/cm^2^ pressure at 30– 45 °C, 45–70 °C, and 70–85 °C for 1 h, respectively. Oil pump was operated to keep the tea tree essential oils circulating in reaction still. Eventually, the cooling water was used to cool the tubes room temperature. The air compressor and nitrogen turn off, and stopping the essential oils circulating. The TTO extracts were separated using 9790 gas chromatograph fitted with a flame-ionization detector. The samples were injected through the split injection port (50:1) onto a 35% diphenyl-65%methylsiloxane polymeride 60 m × 0.25 mm column with a 0.25-μm film. The oven temperature was initially 50 °C for 1 min, and was then increased at 10 °C/min to 250 °C and then held for 9 min. The injector and detector temperatures were maintained at 200 °C and 300 °C, respectively.

### Animals

This study was carried out in accordance with the principles of Yangzhou University, the Institutional Animal Care and Use Committee (SYXK (Su) IACUC 2012–0029). All procedures involving the use of live animals was approved by the Institutional Animal Care and Use Committee. Six mid-lactating Holstein cows were used in this study. All cows were free of clinical signs of disease before isolation of polymorphonuclear leukocytes (PMNL). These cows were fed a TMR to meet 100% of NRC requirements. Cows were milked three times daily at 8:00, 14:00, and 21:00, respectively. The blood (approximately 10 mL) was collected by venipuncture of the jugular vein into acid citrate dextrose anti-coagulant (ACD-A) (Solarbio, Shanghai, China) from each cow before the morning feeding. These tubes were slightly inverted to mix, and placed on ice. Blood samples were immediately transported to laboratory and processed within 30 min of collection.

### Cell culture

Bovine mammary epithelial cells (BMECs) of three mid-lactating Holstein cows were obtained from the Institute of Animal Culture Collection and Application, Yangzhou University. These bovine mammary tissues were digested by collagenase type I (Invitrogen, Shanghai, China) for 3 h, and cells were filtered by nylon mesh (75 μm) to obtain the BMECs. BMECs were seeded in DMEM/F12 medium supplemented with 10% fetal bovine serum (FBS), 4 mm/L glutamine, 1× insulin, transferrin, sodium selenite (10 μg/mL insulin, 5.5 μg/mL transferrin, 0.0067 μg/mL sodium selenite, Invitrogen, Shanghai, China), 15 ng/mL epidermal growth factor (Peprotech, Shanghai, China), 1 μg/mL hydrocortisone, and 4 μg/mL prolactin (Sigma-Aldrich, Shanghai, China).

### Isolation of PMNL

PMNL were isolated and obtained as described previously [19] with minor adaptations. Six mid-lactating Holstein cows were used to isolate the PMNL. The venous blood was transferred to 15 mL sterile tubes containing the 2.2 mL ACD-A, gently inverted to mix, and placed on ice. These tubes were centrifuged at 1000×*g* at 4 °C for 20 min, and the plasma, buffy coat, and one-third of the red blood cells were removed. The remaining cells were transferred to 50 mL sterile tubes and treated with 18 mL of ice-cold deionized water, allowing the tubes to gently invert for no longer than 45 s. Then, the 2 mL 10× PBS solution were added to the tubes to restore the isotonicity conditions, and centrifuged at 1000×*g* at 4 °C for 10 min to remove the supernatant. The pellet was suspended and washed with 20 mL free, calcium- and magnesium-free HBSS solution and centrifuged at 850×*g* at 4 °C for 5 min. Finally, the isolated PMNL were cultured with RPMI-1640 medium supplemented with 5% inactivated FBS and 4 mmol/L glutamine.

### Bacterial strain and culture

*Staphylococcus aureus* (ACTT29213) were obtained from American Type Culture Collection. This strain is isolated from the bovine mastitis. The *S. aureus* were cultured in brain heart infusion (LandBridge, Beijing, China) media at 37 °C and grown for 12–18 h before use.

### Assay of proliferative activity

The proliferative effects of TTO on BMECs was determined using the Cell Counting Kit-8 (CCK-8; Dojindo, Shanghai, China), according to the manufacturer’s protocol. The BMECs (5 × 10^3^ cells/well) were seeded into 96-well plates. After 12 h, BMECs were incubated in DMEM/F12 medium with 0.1% DMSO and either 0, 0.0125%, 0.025%, 0.05%, or 0.1% TTO (*n* = 4) for 6 h. Subsequently, 10 μL CCK-8 reagent was added to cells, followed by incubation at 37 °C, 5% CO_2_ for 2 h. Absorbance at 450 nm was then measured in each well using an auto-microplate reader (Thermo Scientific, Shanghai, China).

### Cell viability assay

BMECs were seeded into 96-well plates (5 × 10^3^ cells/well), and *S. aureus* (5 × 10^3^ CFU/well) added after 12 h. Cells were then cultured in DMEM/F12 medium in the absence of *S. aureus* and TTO (control group), in the presence of *S. aureus* and absence of TTO (*S. aureus* group), and in the presence of *S. aureus* and 0, 0.0125%, 0.025%, 0.05%, or 0.1% TTO (TTO-treated groups) for 6 h (*n* = 4). After incubation, cells were vigorously washed five times with 200 μL of sterile water and incubated with 200 μL DMEM/F12 supplemented with 10 μL of CCK-8 at 37 °C and 5% CO_2_ for 2 h. Absorbance at 450 nm was then measured in each well using an auto-microplate reader. Cell viability (%) = (treatment OD – blank OD)/(control OD – blank OD).

### *S. aureus* growth assay

*S. aureus* were seeded into 96-well plates (1 × 10^5^ CFU/well) in BHI medium supplemented with TTO at concentrations of 0, 0.0125%, 0.025%, 0.05%, or 0.1% TTO (*n* = 3) at 37 °C, 5% CO_2_ for 3, 6, 12, or 24 h, respectively. This was serially diluted and plated on to solid BHI nutrient agar. The number of *S. aureus* was determined by colonies count after 12 h culture at 37 °C.

### *S. aureus* biofilm formation assay

Biofilm formation was evaluated as described previously [20]. Briefly, *S. aureus* were seeded into 96-well plates (2 × 10^6^ CFU/well) and incubated at 37 °C, 5% CO_2_ for 24 h. Supernatants were then removed, BHI medium supplemented with 0 (control group), 0.00625%, 0.0125%, 0.025%, 0.05%, or 0.1% TTO (*n* = 4) added, and plates wrapped with Parafilm and incubated at 37 °C, 5% CO_2_ for 36 h. Next, the plates were rinsed five times with sterile distilled water, and *S. aureus* fixed with 100 μL of methyl alcohol for 15 min, and the supernatant removed. The plates were allowed to dry naturally at room temperature, and then treated with 200 μL 0.1% crystal violet at room temperature for 10 to 15 min. Plates were then rinsed five times, allowed to air dry overnight, and 30% acetic acid in sterile distilled water (200 μL) then added to each well. Plates were incubated with shaking for 1 h, and the solution in each well transferred to a new 96-well plate. To examine *S. aureus* biofilm formation, absorbance at 540 nm was measured using an auto-microplate reader.

### *S. aureus* association and invasion assay

To determine the number of *S. aureus* association to BMECs and *S. aureus* invasion of BMECs at different concentrations of TTO, we followed a previously described method [20]. BMECs grown in 24-well plates were infected using DMEM/F12 medium containing *S. aureus* (1 × 10^5^ CFU/well) at MOI of 1, and treated with 0 (control group), 0.025%, 0.05%, or 0.1% TTO (*n* = 3) at 37 °C, 5% CO_2_ for 3 h. Cells were then washed five times with sterile PBS, and treated with 0.1% Triton X-100, and incubated for 15 min at 37 °C, 5% CO_2_. *S. aureus* association to BMECs were then serially diluted and plated on to solid BHI nutrient agar for colonies count. For the analysis of *S. aureus* invasion of BMECs invasion, BMECs were treated with 250 μg/mL of gentamicin at 37 °C, 5% CO_2_ for 1 h, then washed five times with PBS to remove the gentamicin. Cells were lysed with 0.5% Triton X-100 for 15 min. The remaining live bacteria were then serially diluted, seeded in solid BHI agar plates, and CFUs counted.

### Scanning electron microscope

*S. aureus* (4 × 10^5^ CFU/well) were seeded into the 8-well glass chamber slides in BHI medium, and incubated for 6 h at 37 °C, 5% CO_2_. Then, *S. aureus* were washed three times with 200 μL of sterile PBS, and incubated with 0.05% TTO for 6 h at 37 °C, 5% CO_2_. *S. aureus* without TTO treatment were used as a control. Next, *S. aureus* were washed three times with 200 μL of sterile PBS and fixed with 200 μL 2.5% glutaraldehyde at 4 °C for overnight. Then, *S. aureus* were washed three times with 200 μL of sterile PBS, and successively dehydrated with 30%, 50%, 70%, 80%, 90%, 95%, 100% ethyl alcohol, and 100% ethyl alcohol with Na_2_SO_4_ for 15 min. These slides were dried by automatic critical point dryer (EM CPD-300, Leica, Germany), and these slides surface were gold-plated by high vacuum turbo evaporator (EM SCD500, Leica, Germany). The *S. aureus* samples were observed for SEM (Gemini SEM 300, Carl Zeiss, Germany).

### PMNL chemotaxis assay

The BMECs were seeded into 6-well plates (2.5 × 10^5^ cells/well). After 12 h, the supernatant was removed, and cells washed three times with PBS. BMECs were then incubated in in RPMI-1640 medium (as control group), in the presence of 0.05% TTO and absence of *S. aureus* (TTO-treated group), in the presence of *S. aureus* and absence of TTO (*S. aureus*-treated group), or in the presence of *S. aureus* and 0.05% TTO (TTO and *S. aureus* treated groups) for 6 h (*n* = 3). Then, Supernatants were then filtered through 0.22 μm sterile filters to collect media containing high levels of the chemoattractant, IL-8. Next, 500 μL of medium containing IL-8 was added to the bottom wells of 24-well plates. The chambers (pore size, 5.0 μm; diameter, 6.5 mm; Corning) were placed in the top of wells, and 100 μL PMNL suspension (5 × 10^5^ cells/chamber) were seeded into the chambers. All samples were incubated at 37 °C, 5% CO_2_ for 2 h. Subsequently, chambers were rinsed vigorously three times with 100 μL of CMF-HBSS, and the number of PMNL that had migrated to the bottom wells were determined. For effect of TTO on PMNL chemotaxis, PMNL were seeded into 12-well plates (6 × 10^6^ cells/well), and pretreated with RPMI-1640 medium containing 0.05% TTO (*n* = 5) for 2 h at 37 °C, 5% CO_2_. Next, 600 μL of medium containing a final concentration of 100 ng/mL IL-8 (Peprotech) were added to the bottom wells of 24-well plates. The chambers (pore size, 5.0 μm; diameter, 6.5 mm) were placed in the top of wells, and 100 μL PMNL suspension (6 × 10^5^ cells/chamber) were seeded into the chambers. All samples were incubated at 37 °C, 5% CO_2_ for 2 h. Subsequently, chambers were rinsed vigorously three times with 100 μL of CMF-HBSS, and the number of PMNL that had migrated to the bottom wells were determined.

### Quantitative RT-PCR

For the expression of genes involved in the inflammatory response of BMECs exposed with *S. aureus*, BMECs (2 × 10^5^ cells/well) were seeded in 6-well plates and grown at 37 °C, 5% CO_2_. Cells were divided into four experimental groups as follows: 1) Control, DMEM/F12 medium; 2) TTO treatment, DMEM/F12 medium containing 0.05% TTO; 3) *S. aureus* treatment, DMEM/F12 medium supplemented with *S. aureus*. 4) *S. aureus* and 0.05% TTO treatment, DMEM/F12 medium supplemented with both *S. aureus* and 0.05% TTO treatment. All treatments were incubated for 6 h.

Six mid-lactating Holstein cows were selected to isolate the PMNL for the expression of genes involved in the inflammatory response of PMNL exposed with TTO. PMNL (1.3 × 10^6^ cells/well) were seeded in 24-well plates, and treated with 0% TTO or 0.05% TTO for 2 h (*n* = 6).

After incubation, total RNA was isolated from the cultured cells using a TRIzol kit (Tiangen, Beijing, China). Reverse transcription (RT) was performed using an RT Kit (Takara, Beijing, China). RT reaction mixtures contained 1 μg total RNA and 1 × PrimeScript RT Master Mix in a final volume of 20 μL, and reactions were performed for 15 min at 37 °C. Reverse transcriptase was inactivated by heating to 85 °C for 5 s. qRT-PCR assays were performed using SYBR® Premix Ex Taq™ II Kit (Takara). The qRT-PCR reaction mixture contained 1 × SYBR® Premix Ex Taq™ II, 0.4 μmol/L each forward and reverse primers, and 100 ng cDNA templates in a final volume of 20 μL, and reactions were performed as follows: initial denaturation at 95 °C for 30 s, followed by 40 cycles at 95 °C for 5 s and 60 °C for 30 s. Before the qRT-PCR for samples, the amplification efficiencies of all primers were determined by using standard dilution series. The primers listed in Table 1 are from the previous studies [19, 20]. Efficiency of these primers were determined in the two study. The relative expression of target genes was normalized by the geometric mean of the 2 selected reference genes GAPDH and β-actin and calculated using the 2^−ΔΔCT^ method.

**Table 1:** Primers for real-time PCR analyses^a^

Gene	Primer sequence (5′ to 3′)	Accession number	Size, bp
*GAPDH*	F:GGGTCATCATCTCTGCACCT	NM_001034034	176
	R:GGTCATAAGTCCCTCCACGA		
*IL-6*	F:TCCTTGCTGCTTTCACACTC	NM_173923.2	129
	R:CACCCCAGGCAGACTACTTC		
*IL-1β*	F:CAGTGCCTACGCACATGTCT	NM_174093.1	209
	R:AGAGGAGGTGGAGAGCCTTC		
*TNF-α*	F:GCCCTCTGGTTCAGACACTC	NM_173966.3	192
	R:AGATGAGGTAAAGCCCGTCA		
*TLR-2*	F:CAGGCTTCTTCTCTGTCTTGT	NM_174197.2	140
	R:CTGTTGCCGACATAGGTGATA		
*IL-8*	F:TGGGCCACACTGTGAAAAT	NM_173925.2	136
	R:TCATGGATCTTGCTTCTCAGC		
*κ-casein*	F: CCAGGAGCAAAACCAAGAAC	NM_174294.2	148
	R: TGCAACTGGTTTCTGTTGGT		
*IL-10*	F:TGTATCCACTTGCCAACCAG	NM_174088.1	126
	R:CAGCAGAGACTGGGTCAACA		
*L-selectin*	F:CTCTGCTACACAGCTTCTTGTAAACC	NM_174182.1	104
	R:CCGTAGTACCCCAAATCACAGTT		
*IRAK1*	F: CCTCAGCGACTGGACATCCT	NM_001040555.1	103
	R:GGACGTTGGAACTCTTGACATCT		
*NFKBIA*	F:GGGAGACCTGGCCTTCCTC	NM 001045868.1	101
	R:CCAGAAGTGCCTCAGCGATT		
*TRAF6*	F:AGAACAGATGCCCAATCACTATGAT	NM_001034661.2	100
	R:GTGATTCCTCTGCATCTTTTCATG		
*Lysozyme*	F:GAGAGATGTGAGCTTGCCAGAA	NM_001078159.1	120
	R:TGTAGCTTGTGTGTTGTAATTGCTTTC		
*SOD2*	F:GAGAAGGGTGATGTTACAGCTCAGA	NM_201527.2	100
	R:GGCTCAGATTTGTCCAGAAGATG		
*TLR-4*	F:GACCCTTGCGTACAGGTTGT	NM_174198.6	103
	R:GGTCCAGCATCTTGGTTGAT		
*β-actin*	F:GACCCAGATCATGTTCGAGA	NM_173979.3	145
	R:CTCATAGATGGGCACCGTGT		

^a^Abbreviations: *GAPDH* Glyceraldehyde-3-phosphate dehydrogenase, *IRAK1* Interleukin 1 receptor associated kinase 1, *NFKBIA* NFKB inhibitor alpha, *TRAF6* TNF receptor associated factor 6, *SOD2* superoxide dismutase 2, *TLR-2* Toll like receptor 2, *TLR-4* Toll like receptor 4, *F* Forward; *R* Reverse

### Statistical analysis

Before data analysis, the distribution of normality and homogeneity of variances were studied with a Kolmogorov-Smirnov and Levene test, respectively. If the data were the distribution of normality and homogeneity of variances were equal, statistical analysis was evaluated by One-way analysis of variance (ANOVA), followed by determination of the least significant difference (LSD) for *post-hoc* multiple comparisons of treatment means and tested by the independent sample t-test, using SPSS 19.0 software (SPSS Inc.; Chicago, IL, USA). Otherwise, a Kruskal-Wallis was performed using the non-parametric test for statistical analysis. The gene expression for the 2^−^^ΔΔCt^ and cell viability assays were log_10_ transformed for statistical analysis. *P* values of < 0.05 were considered significant.

## Results

### TTO extracts phytochemical composition

**Fig. 1:**
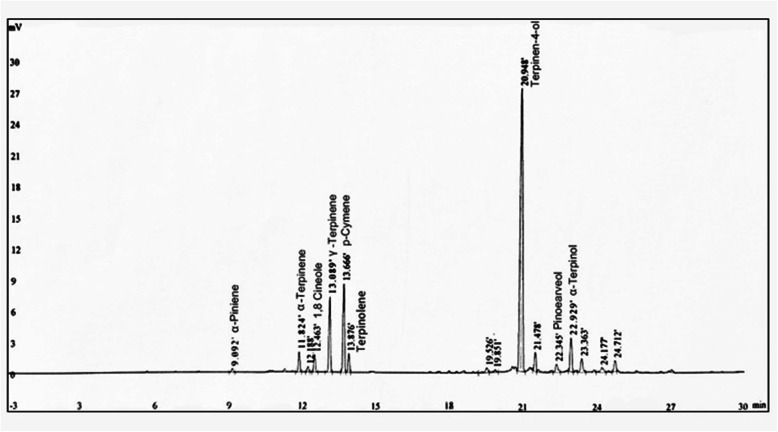
The analysis of TTO extracts major component. Representative chromatogram with the identified major component peaks

The phytochemical composition of tea tree essential oil preliminary product treated by pressure and temperature change in reaction still showed the presence of terpinen-4-ol, α-terpinene, α-terpineol, α-pinene, p-cymene, and 1,8-cineole, γ-Terpinene, terpinolene, and pinocarveol (Fig. 1). According to the New Zealand Standard, concentration of terpinen-4-ol constitutes 30–48% of total effective components in tea tree essential oils preliminary product. Notably, the content of terpinen-4-ol was increased to 60.23% after the preliminary product were treated by pressure and temperature change in reaction still. In addition, TTO extracts have less 0.05% p-cymene which is hazardous for human and animals, compared with p-cymene (1.7%) in New Zealand Standard. In addition, α-pinene, α-Terpinene, γ-Terpinene decreased approximately 4, 8, and 3 folds, relatively to α-pinene (2.1%), α-Terpinene (3%), γ-Terpinene (17.8%) in New Zealand Standard.

### Assessment of the proliferative effects of TTO on BMECs

**Fig. 2:**
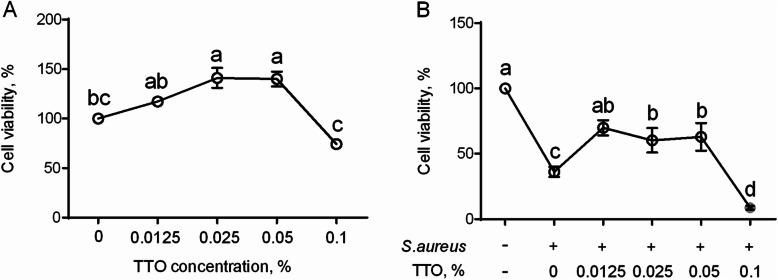
The protective effects of TTO on bovine mammary epithelial cells (BMECs). (**a**) Evaluation of proliferative effects of TTO on BMECs. The BMECs were incubated with 0 (control group), 0.0125%, 0.025%, 0.05%, or 0.1% TTO for 6 h. (**b**) Viability of BMECs exposed to *S. aureus* with or without TTO treatment. BMECs were cultured in the absence of *S. aureus* and TTO (control group), in the presence of *S. aureus* and absence of TTO (*S. aureus* group), and in the presence of *S. aureus* and 0.0125%, 0.025%, 0.05%, or 0.1% TTO (TTO-treated groups) for 6 h. Data are presented as the means ± SEM (*n* = 4). Means at the different concentration of TTO with different letters (**a**-**d**) differ significantly for treatment effect

The relationship between concentration of TTO and their proliferative effect on BMECs was examined by CCK-8 assay. As shown in Fig. 2, results of the proliferative assays demonstrated that concentrations of 0.025% (*P* = 0.02) and 0.05% (*P* = 0.02) TTO were able to increase proliferation of BMECs, compared with the untreated control group (Fig. 2a). In contrast, 0.1% TTO significantly inhibited BMECs viability, relatively to 0.025% and 0.05% concentrations (Fig. 2a). In comparison with controls, BMECs exposed to *S. aureus* exhibited (*P* < 0.001) reduced viability, whereas 0.0125% (*P* = 0.002), 0.025% (*P* = 0.015), and 0.05% (*P* = 0.01) TTO enhanced the viability of BMECs exposed to *S. aureus*, relatively to *S. aureus* treatment alone (Fig. 2b). However, viability of BMECs exposed with *S. aureus* was inhibited at concentration of 0.1% TTO (Fig. 2b). The current results demonstrate that addition of 0.0125%, 0.025%, and 0.05% TTO *in vitro* can promote the viability of BMECs exposed to *S. aureus*.

### Antibacterial activity

**Fig. 3:**
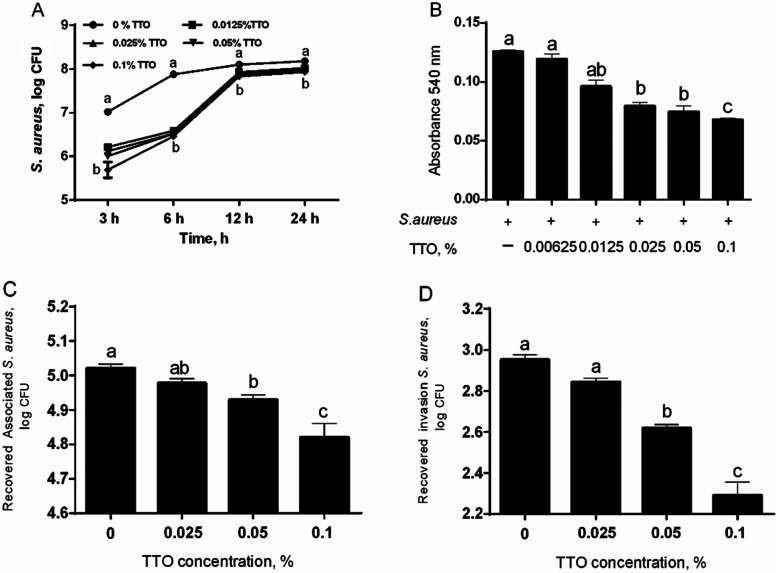
Evaluation of antibacterial effect of TTO. (**a**) The growth inhibition of *S. aureus* during 24 h treatment with 0, 0.0125%, 0.025%, 0.05%, or 0.1% TTO (*n* = 3). Log CFU bacteria were measured after 3 h, 6 h, 12 h, and 24 h. Means at the same time point with different letters (**a, b**) differ significantly for treatment effect. (**b**) Biofilm formation. The *S. aureus* cultures were incubated with 0 (control group), 0.00625%, 0.0125%, 0.025%, 0.05%, or 0.1% TTO (*n* = 6) and absorbance at 540 nm measured. (**c**) The association of BMECs. The BMEC were incubated in DMEM/F12 medium containing *S. aureus* and 0 (control group), 0.025%, 0.05%, or 0.1% TTO (*n* = 3). (**d**) The invasion of BMECs. The BMEC were incubated in DMEM/F12 medium containing *S. aureus* and 0 (control group), 0.025%, 0.05%, or 0.1% TTO (*n* = 3). Data are presented as the means ± SEM. Means at the different concentration of TTO with different letters (a-c) differ significantly for treatment effect

**Fig. 4:**
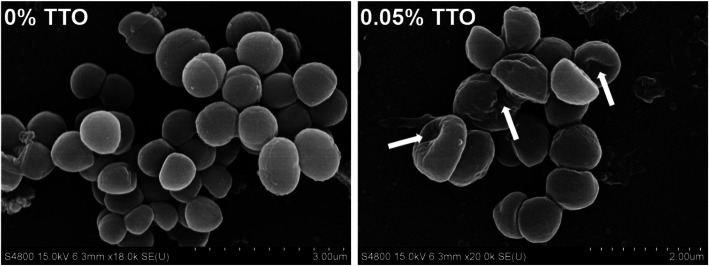
Scanning electron microscopy to evaluate the morphological state of *S. aureus* with TTO. *S. aureus* were incubated with 0 (control) or 0.05% tee trea oil (TTO) for 6 h *in vitro*. White arrowheads indicate *S. aureus* showing a sunken morphology state

The growth of *S. aureus* was reduced at most of TTO concentrations and time points (Fig. 3a). *S. aureus* treated with TTO extracts showed a slow growth by incubation from 3 and 6 h (Fig. 3a). In addition, we also evaluated the effect of TTO on *S. aureus* biofilm formation in present study. Our data showed that at the concentrations of 0.025%, 0.05%, and 0.1% TTO failed to form a *S. aureus* biofilm compared with controls (Fig. 3b). Subsequently, we also investigated the effect of TTO on *S. aureus* association to BMECs *and S. aureus* invasion of BMECs. The present study showed that association of *S. aureus* to BMECs and invasion of *S. aureus* into BMECs were significantly reduced by treatment with 0.05% and 0.1% TTO, compared with control group (Fig. 3c and d). To validate whether addition of TTO have a profound change in the morphological state and structure of *S. aureus*, we observe the morphological state of *S. aureus* by scanning electron microscope (SEM). The *S. aureus* maintains the intact morphology state and structure in the cell surface in the absence of TTO treatment, whereas TTO treatment showed a profound alter in the cell surface, and the *S. aureus* surface become sunk (Fig. 4). These results demonstrated that TTO can reduce the *S. aureus* growth and biofilm formation, and it also prevents invasion of *S. aureus* into BMECs, helping the antimicrobial agents to cure the persistent and chronic infections.

### Effect of TTO on the innate immune response of PMNL

**Fig. 5:**
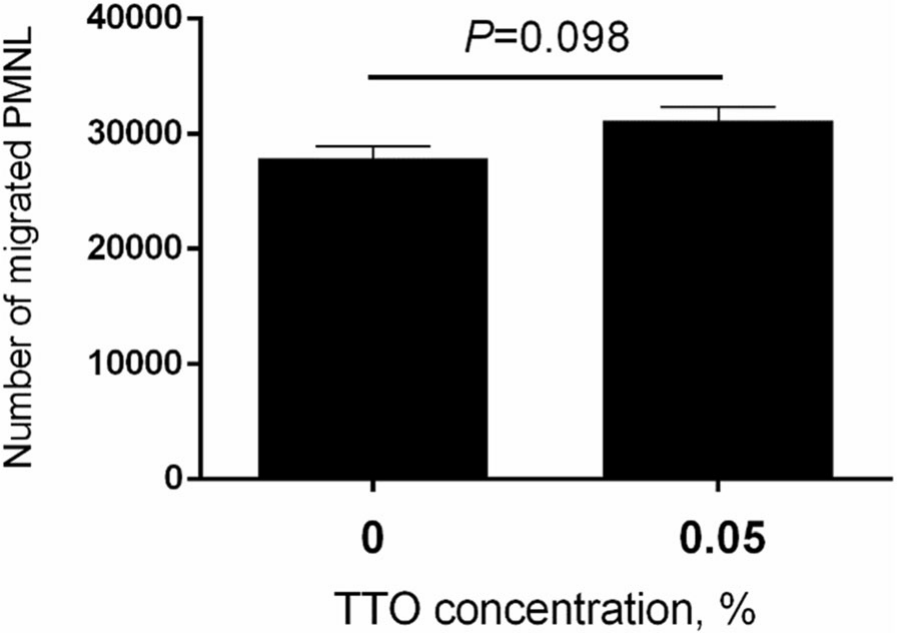
Number of migrated PMNL in response to IL-8 chemokine after PMNL were pretreated with either 0 (control) or 0.05% TTO for 2 h *in vitro*. Data are presented as the means ± SEM (*n* = 5)

The mRNA levels of genes encoding the cytokines, signaling factors, recognition receptor, and pathogen response elements were determined by qRT-PCR (Table 2). Compared with the untreated control group, PMNL treated with 0.05% TTO did not have profound alters (*P* > 0.05) in expression of IL-1β, IL-6, and IL-8. In addition, chemotactic ability of PMNL by IL-8 induction was not changed (*P* = 0.098) after pre-treatment of PMNL with 0.05% TTO (Fig. 5). Notably, IL-10 (*P* = 0.001) and TNF-α (*P* = 0.02) were significantly attenuated in PMNL incubated with 0.05% TTO. For adhesion molecule and pattern recognition receptor, expression of TLR-2, TLR-4 and L-selectin were not changed (*P* > 0.05) by TTO treatment. For these genes involved in NF-κB signal pathway, expression of interleukin 1 receptor associated kinase 1 (IRAKI) was not changed after pre-treatment of PMNL with TTO. Remarkably, NFKB inhibitor alpha (NFKBIA) was significantly attenuated (*P* = 0.02) in PMNL incubated with TTO. For genes related to the killing of pathogens, expression of lysozyme was not affected by the incubation of PMNL with TTO.

**Table 2:** Expression of genes in PMNL incubated with 0 (control) or 0.05% tee trea oil (TTO) *in vitro*

Symbol	Treatment^1^		SEM	*P*-value
	Control	TTO		
*IL-1β*	1.43	0.86	0.23	0.38
*IL-6*	1.19	1.38	0.22	0.81
*IL-8*	1.21	1.25	0.18	0.93
*IL-10*	1.24^a^	0.16^b^	0.18	0.001
*TNF-α*	1.14^a^	0.42^b^	0.46	0.02
*TLR-2*	1.49	0.41	0.47	0.05
*TLR-4*	1.25	0.94	0.13	0.49
*L-seletin*	1.07	1.13	0.11	0.88
*IRAKI*	1.05	0.83	0.12	0.24
*NFKBIA*	1.05^a^	0.52^b^	0.13	0.02
*TRAF-6*	1.04	0.93	0.09	0.51
*Lysozyme*	1.30	0.74	0.21	0.23
*SOD2*	1.16	0.67	0.20	0.14

^a, b^ Means in the same row with different superscripts differ significantly for treatment effect

^1^PMNL from six mid-lactating Holstein cows were incubated in DMEM/F12 medium in the absence of TTO (control group) or in the presence of 0.05% TTO (*n* = 6)

### Effect of TTO on the innate immune response of BMECs exposed with *S. aureus*

**Fig. 6:**
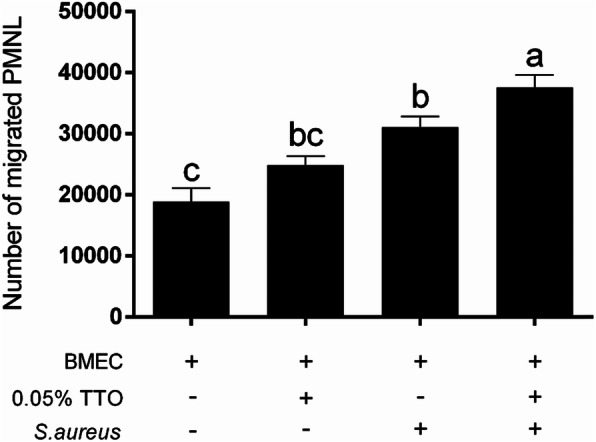
Number of migrated PMNL ing response to high levels of the chemokine IL-8 after BMECs were incubated with 0% TTO (control group), 0.05% TTO, in the presence of *S. aureus* and absence of TTO, or in the presence of *S. aureus* and 0.05% TTO *in vitro*. The media supernatants were from BMECs with different treatment, and added to the bottom wells of 24-well plates. The chambers were placed in the top of wells, and 100 μL PMNL suspension (5 × 10^5^ cells/chamber) were seeded into the chambers. Data are presented as the means ± SEM (*n* = 3). Means at the different treatment with different letters (a-c) differ significantly for treatment effect

The quantitative PCR performance results related to the inflammatory response of BMECs are shown in Table 3. The proinflammatory cytokines IL-1β, IL-6, and TNF-α were significantly downregulated (*P* < 0.001) by the incubation of TTO. Interestingly, addition of TTO enhanced (*P* < 0.001) the expression of IL-8 in BMECs, relatively to untreated control. Remarkably, TTO enhanced the PMNL ability to migrate in *S. aureus*-exposed BMECs, compared with *S. aureus* alone (Fig. 6). In comparison with control, TLR-2 was attenuated by the incubation of TTO (*P* = 0.02), but TLR-4 was not affected. Importantly, TTO did not change the expression of κ-casein in BMECs challenged with *S. aureus* when compared with *S. aureus* treatment alone.

**Table 3:** Expression of genes in BMECs incubated with 0 (control) or 0.05% tea tree oil (TTO) or *S. aureus or S. aureus* with 0.05% TTO *in vitro*

Symbol	Treatment^1^					*P*-value		
	Control	TTO	*S.aureus*	*S. aureus* with TTO	SEM	A	B	A×B
*TLR-2*	1.00	0.46	1.80	0.52	0.25	0.02	0.22	0.28
*TLR-4*	1.00	0.83	0.61	0.64	0.26	0.63	0.06	0.49
*IL-1β*	1.00	0.39	1.25	0.59	0.37	< 0.001	0.02	0.75
*IL-6*	1.00	0.22	1.18	0.59	0.41	< 0.001	0.008	0.25
*TNF-α*	1.01	0.08	0.97	0.14	0.47	< 0.001	0.91	0.51
*IL-8*	1.00	3.69	1.97	3.57	1.26	< 0.001	0.20	0.12
*κ-casein*	1.00	1.48	1.24	1.06	0.25	0.22	0.42	0.02

^1^BMECs were cultured in DMEM/F12 medium in the absence of *S. aureus* and TTO (control group), or in the absence of *S. aureus* and presence of 0.05% TTO, or in the presence of *S. aureus* and absence of TTO, or in the presence of *S. aureus* and 0.05% TTO (*n* = 3). A, TTO treatment; B, *S. aureus* treatment

## Discussion

The bovine mammary epithelium is the first line of defense against invasion of pathogen bacteria, and is vital role in the innate immune response to alleviate the severity of infections [21]. The mammary epithelium infected with pathogen elicits the release of inflammatory mediators, mounting the innate immune responses to enhance the resolution of pathogenic bacterial infection [22]. However, the inflammatory response of mammary epithelium caused by *S. aureus* infection is usually slow, and the infection frequently becomes persistent [23]. The adhesion of *S. aureus* to mammary epithelial cells is as initial step in pathogenesis [24]. The adhesion is associated with the non-specific or specific binding between bacterial cell-associated ligands and host cell surface receptors, and there is strong evidence that invasion of mammary epithelial cells by bacterium is related to an endocytic process, allowing the interaction between bacteria and host cells via surface proteins [24]. Persistent and chronic infections caused by *S. aureus* is a serious problem due to *S. aureus* invasion into BMECs, and only extracellular bacteria is treated by antimicrobial agents, but *S. aureus* within cells is protected from antimicrobials [25]. In addition, the biofilm matrix is a complex mixture of macromolecules containing exopolysaccharides, proteins and DNA that bind to the bacterial surface [26]. The surface biofilms are resistant to antibiotics treatment and as effective barrier against the immune cells response, and actively response to some persistent and chronic infection [27, 28]. In present study, the phytochemical composition of TTO extract exhibit the presence of terpinen-4-ol, α-terpinene, α-terpineol, α-pinene, p-cymene, and 1,8-cineole, γ-terpinene, terpinolene, and pinocarveol. Remarkably, the concentration of terpinen-4-ol involved in the antibacterial activity [12] contains approximately 60.23%. The phytochemical composition of p-cymene involved in the hazards for human and animals show less 0.05% concentration. These results indicate that TTO extracts exhibited a positive role for the antibacterial properties and safer for human and animals. To our knowledge, no study has investigated the effect of TTO on *S. aureus* biofilm formation, *S. aureus* association to BMECs and invasion of *S. aureus* into BMECs. Therefore, the development of alternative, nontoxic agents for the resolution bovine mastitis triggered by *S. aureus* are urgent need for improving milk quality and human health.

TTO has beneficial role in antibacterial activity and anti-inflammatory properties [12]. Our results demonstrated that 0.025% and 0.05% TTO were able to increase proliferation of BMECs. Previous study reported that 10% TTO is able to inhibit the proliferation of melanoma cells and overcoming the resistance to multidrug [29]. Our results indicate that low concentration of TTO did not impair the BMECs function, and may promote the innate immune response of BMECs to be effective role for the resolution of bovine mastitis. Notably, 0.0125%, 0.025%, and 0.05% TTO significantly enhanced the viability of BMECs exposed to *S. aureus*, compared with *S. aureus* treatment alone, suggesting that TTO may be able to mount the innate immune responses to reduce the *S. aureus* damage to BMECs. In this process, TTO exerts a positive role by reducing the effects of *S. aureus* on the BMECs, but not by moderating effects of BMECs. For antibacterial activity, addition of TTO decreases the *S. aureus* growth and biofilm formation, indicating that TTO have a positive role in preventing and controlling biofilm formation strategy in the context of bovine mastitis caused by *S. aureus*. The association of *S. aureus* to BMECs and invasion of *S. aureus* into BMECs were significantly reduced by treatment with 0.05% and 0.1% TTO, suggesting that TTO can promote and help the antimicrobial agents to cure the persistent and chronic infections caused by *S. aureus*. During the bovine mastitis, the bacteria often attacks the mammary epithelial cells and impairs cells function that fail to solve the immune response against the bacterial infection [24], whereas TTO is probably effective role in mediating the protective immune response of BMECs.

During bovine mastitis, circulating blood PMNL were actively recruited to mammary gland infected by pathogen for alleviating the inflammatory response and elimination of pathogen [18, 30], and eventually these PMNL migrated to mammary gland become the predominant somatic cells in milk [31]. Therefore, PMNL play a decisive role for resolution of mastitis. The recruitment process of PMNL is controlled by chemoattractants, such as IL-8, C-C motif ligand, and C-X-C motif ligand chemokines released by resident immune cells and mammary epithelial cells [20]. In the current study, chemotactic ability of PMNL by IL-8 induction was not altered after pre-treatment of PMNL with 0.05% of TTO. During the bacterial infection, PMNL can trigger the profound changes in the adhesion molecules for the resolution of pathogenic bacteria. Toll-like adhesion molecule and pattern recognition receptors can activate the NF-κB proinflammatory signal pathways that respond to the activation of IRAKI and TRAF-6, and further induce the IKK complex to trigger the phosphorylation of IκB and the release of NF-κB. Subsequently, NF-κB binds to the TNF-α, IL-6, and IL-1β promoter, leading to their elevation in transcriptional level [32]. To date, no studies have investigated the effect of TTO on the innate immune responses of PMNL. The TLR-2 and TLR-4 recognize the gram-positive and gram-negative bacteria, respectively [33]. Our results show that mRNA level of TLR-4 was not changed after pre-treatment of PMNL with TTO, but TLR-2 was significantly attenuated in PMNL treated with TTO. Previous study has demonstrated that plant essential oils extract did not have a positive effect against the Gram-negative bacteria [34]. Therefore, TTO modifies the innate immune responses of PMNL by an attenuation in the TLR-2 rather than to TLR-4. In addition, PMNL treated with 0.05% TTO have no profound changes in the expression of TRAF-6 and IRAKI. The NFKBIA encoding the IκBα gene was decreased in PMNL with 0.05% TTO. This downregulation of NFKBIA is probably involved in an attenuation in the NF-κB expression because NF-κB is widely accepted to bind the genes promoter to induce its transcription, and ultimately leading to the overall decrease in the mRNA level of proinflammatory genes. Consistently, our results also show the evidence that TNF-α was attenuated after PMNL treated with TTO, indicating that TTO has a positive role for relieving the inflammatory response.

The BMECs trigger the activation of innate immune responses, and form the first line of defense against invasion of pathogen [21]. During the chronic infections triggered by *S. aureus*, mammary gland actively exerts the protective immune response by chemokines and adhesion molecule induced by mammary epithelial cells. The expression of TLR-2 gene involved in the innate immune response was downregulated by TTO treatment in BMECs challenged with *S. aureus* compared with *S. aureus* alone, however, TLR-4 was not affected. Remarkably, IL-1β, IL-6, and TNF-α were attenuated by incubation with 0.05% TTO in *S. aureus*-exposed BMECs, relatively to *S. aureus* alone, suggesting that TTO can alleviate the proinflammatory responses effect on *S. aureus*-exposed BMECs. Interestingly, *S. aureus*-exposed BMECs with TTO treatment increased the IL-8 expression. Consistently, 0.05% TTO enhanced the PMNL ability to migrate in *S. aureus*-exposed BMECs compared with *S. aureus* alone and control because increased the synthesis of IL-8 known for PMNL chemotactic function. This finding suggests that blood PMNL migration to mammary cells was elevated to promote the elimination of *S. aureus* and resolution of bovine mastitis when treatment with TTO induces the expression of IL-8 in bovine mammary gland infected by *S. aureus*. To evaluate the safety of TTO extracts for application in bovine *in vivo*, measurement of a casein mRNA level was determined to evaluate the cytotoxic effects of TTO on BMECs. Our result demonstrated that 0.05% TTO did not have a profound alter in all experimental groups. Besides, addition of TTO can reduce the *S. aureus* growth, *S. aureus* biofilm formation and *S. aureus* invasion of BMECs. These result indicates that TTO may be beneficial for prophylactic mastitis and anti-inflammatory and is likely to be safe for *in vivo* use in bovine.

## Conclusions

The present study indicates that 0.025% and 0.05% concentration of TTO increased the proliferation of BMECs, and enhanced the viability of BMECs exposed to *S. aureus*. BMECs incubated with TTO can relieve the inflammatory responses triggered by *S. aureus*, and enhance IL-8 expression to recruit the PMNL to mammary gland to mount the innate immune response of BMECs and PMNL. Importantly, TTO did not affect the expression of κ-casein in *S. aureus*-exposed BMECs. Therefore, TTO should be further considered for use as a novel therapy strategy for the bovine mastitis caused by *S. aureus*, to improve milk production and quality.

## Data Availability

All data generated or analysed during this study are available from the corresponding author by request. The datasets supporting the conclusions of this article are included in the article.

## Abbreviations

BMECs: Bovine mammary epithelial cells
FBS: Fetal bovine serum
GAPDH: Glyceraldehyde-3-phosphate dehydrogenase
IRAK1: Interleukin 1 receptor associated kinase 1
NFKBIA: NFKB inhibitor alpha
PMNL: Polymorphonuclear leukocy
*S. aureus*: *Staphylococcus aureus*
SOD2: Superoxide dismutase 2
TLR-2: Toll like receptor 2
TLR-4: Toll like receptor 4
TRAF6: TNF receptor associated factor 6
TTO: Tea tree oil
